# Metaverse-Powered Experiential Situational English-Teaching Design: An Emotion-Based Analysis Method

**DOI:** 10.3389/fpsyg.2022.859159

**Published:** 2022-03-24

**Authors:** Hongyu Guo, Wurong Gao

**Affiliations:** ^1^School of Foreign Languages, Zhejiang Gongshang University, Hangzhou, China; ^2^Graduate School of Education, University of Perpetual Help System DALTA, Metro Manila, Philippines

**Keywords:** metaverse, neural networks, EEG, crowd-creation, emotion recognition

## Abstract

Metaverse is to build a virtual world that is both mapped and independent of the real world in cyberspace by using the improvement in the maturity of various digital technologies, such as virtual reality (VR), augmented reality (AR), big data, and 5G, which is important for the future development of a wide variety of professions, including education. The metaverse represents the latest stage of the development of visual immersion technology. Its essence is an online digital space parallel to the real world, which is becoming a practical field for the innovation and development of human society. The most prominent advantage of the English-teaching metaverse is that it can provide an immersive and interactive teaching field for teachers and students, simultaneously meeting the teaching and learning needs of teachers and students in both the physical world and virtual world. This study constructs experiential situational English-teaching scenario and convolutional neural networks (CNNs)–recurrent neural networks (RNNs) fusion models are proposed to recognize students’ emotion electroencephalogram (EEG) in experiential English teaching during the feature space of time domain, frequency domain, and spatial domain. Analyzing EEG data collected by OpenBCI EEG Electrode Cap Kit from students, experiential English-teaching scenario is designed into three types: sequential guidance, comprehensive exploration, and crowd-creation construction. Experimental data analysis of the three kinds of learning activities shows that metaverse-powered experiential situational English teaching can promote the improvement of students’ sense of interactivity, immersion, and cognition, and the accuracy and analysis time of CNN–RNN fusion model is much higher than that of baselines. This study can provide a nice reference for the emotion recognition of students under COVID-19.

## Introduction

The metaverse does not seem to have a definition that everyone agrees with. To the best of our knowledge, as a new concept, the metaverse is gradually formed based on 5G, virtual reality (VR), and other technologies (Shen et al., 2021; Sparkes, 2021; Jeong et al., 2022). Metaverse is the whole internet on VR and augmented reality (AR) glasses. VR and AR glasses are the next-generation mobile computing platform to be popularized, and metaverse is the presentation of the internet industry on this new platform. From this perspective, the scope of the metaverse is very broad, which includes social networking, e-commerce, education, games, and even payment (Pesce, 2021). All kinds of Internet applications we are familiar with today will have their own presentation in the metaverse.

Metaverse is a key trend to integrate artificial intelligence (AI), VR, blockchain, and other technologies to provide students with a virtual learning field (Foster and Shah, 2021; Herrera-Pavo, 2021) therefore, this paper focuses on the field of English education. The initial prototype of the metaverse is the virtual network world formed in the era of transformation from 2D to 3D technology. Therefore, the initial model of the metaverse has the characteristics of the virtual world. Some scholars have proposed that the real immersion of the virtual world has four technical characteristics, such as audiovisual, interactivity, persistence, and immersion (Smith and Mulligan, 2021). In particular, users can motivate their perception through vision and hearing in the virtual world and interact with other users. Finally, users can have strong telepresence through the integration of multi-perception devices. In different media forms, there have been many prototypes of metaverse with these four characteristics, providing innovative attempts for the construction of metaverse at different levels.

At present, most of the technologies or products that embody the metaverse are mainly limited to the field of electronic entertainment, but simply treating the metaverse itself as a video game is obviously a superficial interpretation, which will make people ignore the great potential of the metaverse for English education. It is of great theoretical and practical significance to explore the reform opportunities that may be brought by the in-depth application of the metaverse, and the form and technical realization of the educational metaverse, and closely combine a philosophical digital survival and game learning (Allen and McLaren, 2021; Wiederhold, 2021; Rospigliosi, 2022).

The metaverse of English teaching can be understood as the educational application of the metaverse, so it creates digital identities for teachers and students, opens up formal and informal teaching places in the virtual world, and allows teachers and students to interact in the virtual teaching places. Considering the metaverse from the perspective of educational philosophy, it can be found that its most prominent enabled advantage is to create an immersive teaching interaction field for teachers and students. The field of English-teaching metaverse breaks through the limitations of the physical world and creates a new virtual education world through the network teaching space so that teachers and students can meet the needs of real and virtual teaching in the physical and virtual world at the same time. However, the virtual world in the metaverse of English teaching is neither a simple copy of the physical world, nor the “parallel universe” of another physical world, but a redevelopment of the physical world. Its media-enabled characteristics can compensate for the shortcomings of the physical world and even surpass the limitations of the physical world in some dimensions to form a special educational metaverse field, which can play an overall presence effect.

The emotional state of humans can be identified by non-physiological indexes such as facial expression and body movement, and also physiological indexes such as blood pressure and electroencephalogram (EEG) (Ilyas et al., 2021). Since non-physiological indexes are subjective and can hide from others through training, and people with autism and limb dysfunction cannot express their emotions independently, it is limited to judge the emotional state through non-physiological indexes. Judging emotional state through physiological indexes has higher reliability and stronger representativeness. EEG contains rich information and convenient collection. As the basis for judging emotional physiological indexes, it is welcomed by more and more researchers. This paper designs an experiential English-teaching scene, analyzes the collected students’ EEG, and performs personalized English teaching for students by analyzing their emotions.

Most studies on emotion recognition are focused on the single convolutional neural networks (CNN) model (Keelawat et al., 2021). Although CNN has a strong extraction ability of frequency domain and spatial domain features, its analysis ability of time-domain features is relatively weak. However, time-series signals such as EEG contain a certain amount of time-domain information. It is inevitable for CNN of single structure to miss part of the time-domain information of EEG, which makes that the accuracy of CNN based EEG emotion recognition method still has a great space for improvement. Whereas the gated recurrent unit (GRU) in recurrent neural network (RNN) (Banerjee and Singh, 2021) is good at processing time-domain information; thus, this paper constructs the model based on GRU and RNN.

The main contributions of this paper are summarized as follows: (i) The experiential situational English-teaching scenario is constructed. (ii) CNN–RNN fusion model is proposed to recognize students’ emotion EEG in experiential English teaching during the feature space of time domain, frequency domain, and spatial domain. The rest of this paper is organized as follows. Section “RELATED WORK” proposed the related work. In Section “EXPERIMENT AND ANALYSIS,” the CNN–RNN fusion model is constructed. The experimental results are shown in Section “EXPERIMENT AND ANALYSIS.” The conclusion of this paper is given in Section “CONCLUSION.”

## Related Work

Although the metaverse has not been widely concerned by educational researchers, its underlying technology has been discussed by scholars and achieved many results. In Yang et al. (2020), by establishing an AI education robot based on voice interaction, the authors built a hybrid physical education teaching mode to realize the personalized education of students. In Wang (2021), the author proposed a simulation model which could dynamically analyze the role of students in educational management services. In Xuan et al. (2021), the authors presented a deep neural network-based personalized learning material recommendation algorithm. In Xiao and Yi (2021), the authors used AI to perform personalized education reform, analyze the information of students before entering colleges and universities, and propose a construction method of personalized AI-based training model. Based on the theory of deep learning, in Wang Z. et al. (2021), the authors evaluated the practical application value of teaching methods under the guidance of educational psychology and AI design. In Sun et al. (2021), the authors combined the AI module with knowledge recommendation and developed an online English-teaching system based on the general teaching assistant system.

The digital twin also provides a resource pattern for the integration of virtual and real education. To improve the teaching efficiency of rhythmic gymnastics, based on VR image recognition technology and digital twin, combined with the actual needs of rhythmic gymnastics teaching, in Shi (2021), the author constructed the corresponding auxiliary teaching system. For the sake of studying the methods to improve teachers’ teaching ability, in Chen et al. (2021), the authors established a machine learning and digital twin-based corresponding teacher ability evaluation model. In Razzaq et al. (2022), the authors proposed a digital twin framework called “deep class rooms” to monitor school attendance and curriculum content.

In the most critical aspect of creating the telepresence, some researchers have carried out research on the effect of VR teaching and verified the role of VR technology in promoting students’ learning participation, teaching efficiency, and learning effect through experiments. At the same time, it also provides relevant references for students to create a virtual environment with telepresence. In Tang (2021), the author studied the application of VR technology in college physical education and discussed the role of VR technology in improving the quality of physical education. In Young et al. (2020), the authors evaluated students’ experience of applying immersive technology in a higher education environment, especially in physical geography students’ exploration of geomorphology theory using VR. Based on the VR education platform, in Zhang et al. (2020), the authors clarified the importance and some disadvantages of education under the traditional education mode. Through the collected literature and survey data, the authors analyzed the feasibility of the combination of VR and education and provided a theoretical basis for it. In Hagge (2021), VR technology was introduced into four face-to-face geography courses in two semesters. Throughout the semester, part of the students regularly used VR devices to visit places related to the lecture.

Many EEG emotion recognition methods have been proposed. In Liu and Fu (2021), the authors proposed to learn multi-channel features from EEG signals for human emotion recognition. In Aguinaga et al. (2021), the authors proposed a two-stage deep learning model to recognize emotional states by associating facial expressions with EEG. In Song et al. (2020), the authors proposed a dynamic graph CNN-based multi-channel EEG emotion recognition method. In Zeng et al. (2021), the authors proposed an emotional wheel attention-based emotion distribution learning model (EWAEDL). In Gao et al. (2022), the authors proposed a novel refined-detrended fluctuation analysis method, that was, multi-order detrended fluctuation analysis (MODFA). In Han et al. (2021), the authors proposed a novel cross-modal emotion embedding framework, called EmoBed, which aimed to improve the performance of existing emotion recognition systems by using the knowledge from other auxiliary patterns.

## Methodology

In this section, the CNN–RNN fusion model is proposed to recognize students’ emotion EEG in experiential English teaching during the feature space of time domain, frequency domain, and spatial domain.

This paper proposes a fusion network of CNN and RNN. Using the good spatial expansion performance of CNN, its neuron and feature convolution operation can effectively extract information in the frequency domain and spatial domain. GRU in RNN can be used to describe the output of continuous state in time and has memory function through time expansion and calculation of neuron and multiple time outputs. Therefore, the combination of CNN and GRU can simultaneously obtain information features in the frequency domain, spatial domain, and time domain, and the extracted EEG feature information is more comprehensive.

**FIGURE 1:**
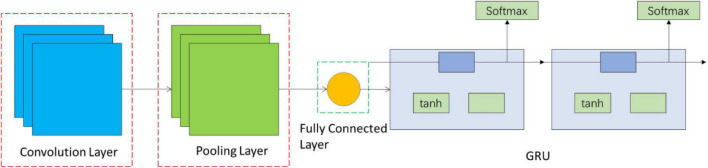
CNN–RNN fusion network structure.

The CNN–RNN fusion network structure in this method is shown in Figure 1. The fusion network is composed of CNN and RNN, wherein CNN is composed of three convolutional layers, three pooling layers, and one fully connected layer. The two-layer RNN connection is behind the fully connected layer of CNN. The fusion network not only retains the frequency-domain and spatial processing capability of CNN but also increases the interpretation of RNN in time series.

### Emotion Electroencephalogram Data Preprocessing

Due to the weakness of EEG signals, the data acquisition process is inevitably interfered with by noise such as electrooculogram (EOG), electromyography (EMG), and head movement. Therefore, in the first step of EEG analysis, EEG data preprocessing should be performed to remove the noise and artifact mixed in the signal, so as to reduce the influence of the noise background as much as possible and improve the signal to noise ratio. The data preprocessing process in this study includes three steps: band-pass filtering, EOG removal, and effective data interception.

Since electromagnetic and power frequency interference often occurs in a high-frequency band, the artifact noise is usually distributed in low-frequency band, whereas the main component of EEG frequency of normal adults is concentrated below 45 Hz, so the band-pass filter of 1–45 Hz is used to filter out the frequency band that is easy to cause interference and retain the main frequency band. The experiment in this study both involved visual-evoked potential, and the blinking and eye movement of the subjects is inevitable, so the EEG signals are mixed with a lot of EOG interference. Independent component analysis (ICA) is a blind source separation method, which can avoid the loss of the main components of the signal and has an ideal denoising effect on the signal. Therefore, ICA is used to remove the EOG artifacts.

The principle of ICA analysis is to decompose the observed signals into several independent source components (Liu et al., 2021) through the optimization algorithm in accordance with the principle of statistical independence. Theoretically, it is considered that the collected EEG signals can be regarded as the mixed signals of interference signals (artifact, EMG, electromyography, etc.) and EEG signals generated by different independent sources. ICA decomposed EEG signals and artifact signals into a series of mutually independent source components. Through certain judgment criteria, the artifact interference mixed in EEG signals is removed, and the useful EEG component (Maziero et al., 2021) is retained. Finally, the useful original EEG signals are restored through an inverse operation.

The collected EEG data contain the information of each acquisition channel and the corresponding EEG values at different moments, which are preprocessed as the input data of the fusion network.

### Network Initialization

Due to the difference in each channel, the value of the collected EEG data of students varies greatly. To improve the accuracy of training, the sample EEG data are normalized and then divided into training set, verification set, and test set according to the ratio of 6:2:2. The sample labels are converted into binary representation using one-hot encoding (Al-Shehari and Alsowail, 2021). The size of the three-layer convolution kernels is three and the step size is one. Rectified linear unit (ReLU) activation function is used to prevent the vanishing gradient, and the nonlinear of the network is added to make the network training faster (Ma et al., 2021). The kernel size of the max-pooling layer is 2, and the max-pooling layer has the feature of translation invariance, which can reduce the parameters for dimension reduction while retaining the main features to prevent overfitting. The activation function of the GRU layer uses the hyperbolic tangent function tanh, and the output mapping is between −1 and 1. The output range is limited and the optimization is stable.

### Feature Extraction and Emotion Analysis

The convolution operation is performed on the input matrix data that is, the inner product operation is performed through the matrix with a certain weighted convolution kernel. The output after the inner product is one of the frequency-domain features. Due to a large number of convolution kernels, various frequency-domain features will be extracted after convolution. In the process of convolution, the local correlation of image space is used to automatically extract the features of input data. Meanwhile, max pooling is used to reduce the number of parameters and accelerate the calculation process after each level of convolution.

After several times of convolution and pooling, more detailed spatial features in the frequency domain are extracted and used as input data of the GRU network. The update gate output of GRU is close to 0, indicating that some information of the hidden state of the previous layer is forgotten in the hidden layer, and close to 1 means that the hidden layer continues to be retained. When the reset gate of the GRU is close to 0, it indicates that certain information at the last moment is forgotten in the current memory; when the reset gate is close to 1, it indicates that the information continues to be retained in the current memory. Then, the two parts of information are added and normalized between −1 and 1 through the Tanh activation function. Therefore, the memory content of this moment is composed of two parts: one is the reset gate to store important information related to the past and the other is to add important information input at the current moment. These two parts constitute all the memory content of the current time and extract the time-domain features.

After extracting time-domain information from EEG with frequency-domain and spatial features through a two-layer GRU unit, the Softmax activation function is used to output emotional states, and the output emotional states are compared with labels, and then, reverse calculation is performed. After repeating many times, the identified emotional states are output.

## Experiment and Analysis

### Experiential English-Teaching Scenario Construction

**FIGURE 2:**
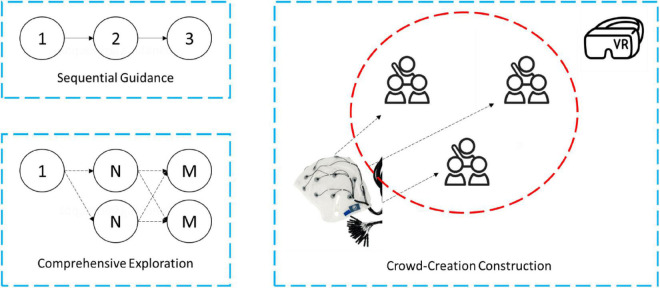
The architecture of experiential English-teaching scenario.

According to the characteristics of technological interaction, students in experiential English-teaching scenario is designed into three types: sequential guidance, comprehensive exploration, and crowd-creation construction. Among them, sequential guidance refers to the interactive guidance in which students understand and complete the knowledge of interactive modules step-by-step according to the task prompts of the environment. Comprehensive exploration is an inquiry learning method based on sequential guidance and free exploration. Crowd-creation construction refers to the collaborative work of students in groups, whose main task is to complete the construction of space by utilizing the modular construction function provided by the environment, so as to promote the development of their collaborative ability and inquiry ability. The architecture of the experiential English-teaching scenario is shown in Figure 2.

Electroencephalogram analysis was performed using OpenBCI EEG Electrode Cap Kit, which is engineered for reliable EEG biopotential measurements with wet electrodes. In terms of EEG data analysis, a single-channel signal monitor that is not easy to be interfered with is adopted because the students need to wear VR glasses. The main body of the monitor is fixed by a band, and four EEG waves are obtained by using the upper frontal electrode and ear electrode, and time-domain characteristics and time-frequency characteristics of the obtained signals are analyzed.

For the collected multidimensional data, due to its different dimensions, it is difficult to use a consistent standard to judge, so the entropy method is used for mining and analysis (Wang L. et al., 2021). The entropy method regards the object as a system and determines the entropy *E* of the system according to the probability *p*_i_ of each state in the system, as shown in Equation (1). The lower the entropy, the higher the stability.


(1) $$E=\sum_{i=1}^{N}p_{i}\,\ln p_{i}$$

At the same time, the similarity of students’ behaviors can be calculated by integrating the atomic economic algorithm and cosine algorithm, as shown in Equations (2) and (3). In Equation (2), AE is the original atomic economic value, mw is the molecular weight, and *AE* is the percentage of the ratio of the mw produced by English teaching to the original input of ∑*mwe*. In Equation (3), (*a*_*1*_, *a*_*2*_) is the atomic economic value vector *AE*_*1*_ of the first class, and (*b*_*1*_, *b*_*2*_) is the atomic economic value vector *AE*_*2*_ of the second class. The included angle cosine represents the similarity of students’ behavior data. The closer the value is to 1, the more similar the two kinds of learning behaviors are.


(2) $$A\,E=\frac{m\,w}{\sum m\,w\,e}\times 100\%$$

(3) $$\cos\alpha =\frac{a_{1}\,a_{2}+b_{1}\,b_{2}}{\sqrt{a_{1}^{2}+b_{1}^{2}}\,\sqrt{a_{2}^{2}+b_{2}^{2}}}$$

The analysis of EEG data can find the changes in the cognitive load of students. This paper classifies cognitive engagement that is, the value of cognitive engagement within the range of 0–100 is divided into seven levels (11–24, 25–38, 39–51, 52–64, 65–78, 79–91, and 92–100), and the median value of the seven levels is 52–64. The range of cognitive engagement is defined as shown in Equation (4), where B represents the average value of the input coefficient.


(4) $$C\,E=\left\{ \begin{array}{cc} \mathrm{High}\,\mathrm{Cognitive}\,\mathrm{Engagement}\;\text{B}\geq 61 & \\ \mathrm{Medium}\,\mathrm{Cognitive}\,\mathrm{Engagement}\;51\leq \text{B}\leq 60 & \\ \mathrm{Low}\,\mathrm{Cognitive}\,\mathrm{Engagement}\;\text{B}\leq 50 & \end{array}\right.$$

### Simulation Results and Discussion

#### Utility Analysis of Learning

The collected EEG data of students were converted into AE values to calculate its entropy values. The comparison results of experiential English teaching’s utility entropy are shown in Tables 1–3.

**TABLE 1:** Interactive utility.

	Interactive utility			Comprehensive assessment
	Hardware	Software	Behavior intention	
Sequential guidance	3.8012	2.6645	2.6719	8.1516
Comprehensive exploration	2.5674	2.0312	2.3415	5.2615
Crowd-creation construction	2.9546	2.7108	2.2202	6.8901

**TABLE 2:** Immersion utility.

	Immersion utility			Comprehensive assessment
	Interactivity	Immersion	Telepresence	
Sequential guidance	2.8302	2.2002	1.9265	5.9600
Comprehensive exploration	2.1496	1.4869	1.5947	6.9133
Crowd-creation construction	2.0560	1.3777	1.7245	5.0589

**TABLE 3:** Cognitive utility.

	Cognitive utility			Comprehensive assessment
	Emotional information	Knowledge construction	Flow experience	
Sequential guidance	1.9920	1.3784	0.7990	4.9652
Comprehensive exploration	2.6252	2.3456	1.4197	5.2601
Crowd-creation construction	1.0506	1.4873	1.6162	4.0550

According to the comparison of entropy value of English-teaching utility, it can be found that interactive utility is higher than immersion utility (8.1516 > 5.9600), and immersion utility is higher than cognitive utility (5.9600 > 4.9652). This may be due to the fact that the students are most likely to be shocked by the interaction and immersion experience brought by the metaverse environment when they first touch it, and it has not completely reached the level of promoting cognitive development. In the comparative analysis of the three types of activities, the highest is the comprehensive exploration activity (*E*_*2*_ = 20.1103), followed by the sequential guidance activity (*E*_*1*_ = 19.0768) and finally the crowd-creation construction activity (*E*_*3*_ = 16.0040). This indicates that compared with the comprehensive inquiry activity, the sequential guidance activity is too restrictive and the crowd-creation construction activity is too open. A comprehensive exploration can not only give the students guidance but also give them certain free exploration space.

#### Eigenvalue Analysis of Cognitive Engagement

Students’ cognitive engagement was obtained by EEG data analysis. The EEG monitor was used to detect the brain waves of students in four states (calm state, sequential guiding state, comprehensive exploration state, and crowd-creation construction state). The sampling rate was 1,000 Hz, and the mean variation coefficients of five brain waves (δ, θ, α, β, and γ waves) were obtained.

As can be seen from Table 4, the average input coefficient of students in the four states shows a changing trend from low to high, that is, *B*_*1*_ = 54.3012, *B*_*2*_ = 59.8749, *B*_*3*_ = 62.0576, and *B*_*4*_ = 74.8327. According to the degree of cognitive engagement, calm state and sequential guided learning state are at the level of “medium cognitive engagement” (51 ≤ B ≤ 60), whereas comprehensive exploration and crowd-creation construction learning state are at the level of “high cognitive engagement” (B ≥ 61). This indicates that in the experiential English-teaching metaverse framework designed in this study, three kinds of learning activities supported the cognitive development of the students from “medium cognitive involvement” to “high cognitive involvement.”

**TABLE 4:** The variation coefficient of EEG eigenvalue and the mean value of input coefficient.

	δ wave (mV)	θ wave (mV)	α wave (mV)	β wave (mV)	γ wave (mV)	B
Calm state	142.6108	39.8990	13.5547	9.1654	4.9607	54.3012
Sequential guidance	221.2198	68.8475	20.7365	15.0200	5.6347	59.8749
Comprehensive exploration	216.8784	65.0997	16.6875	17.7402	4.4742	62.0576
Crowd-creation construction	136.3233	31.7755	10.5336	12.8006	11.4253	74.8327

#### Emotion Analysis Accuracy and Emotion Analysis Time

In addition, the vital metrics of emotion analysis such as the emotion analysis accuracy and emotion analysis time were used for comparison. The experiment was driven on the SEED-IV dataset, using different movie clips as a library of four emotions (peace, sadness, fear, and happiness) for a total of 15 participants. EWAEDL (Zeng et al., 2021), MODFA (Gao et al., 2022), EmoBed (Han et al., 2021), and the proposed method were compared on the SEED-IV dataset.

**FIGURE 3:**
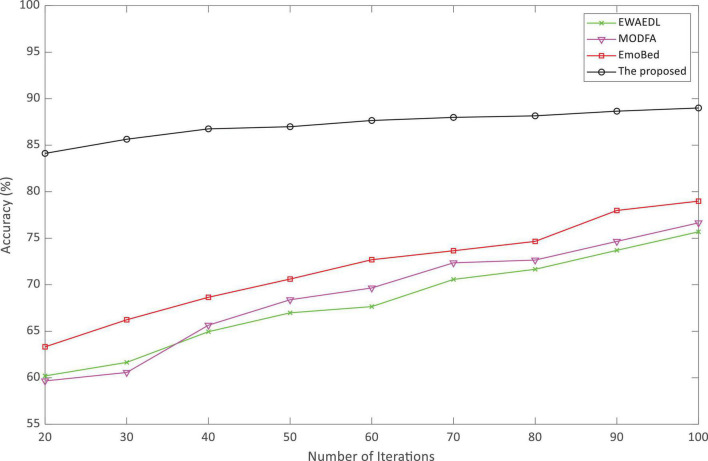
Accuracy of the experiential English-teaching emotion analysis.

**FIGURE 4:**
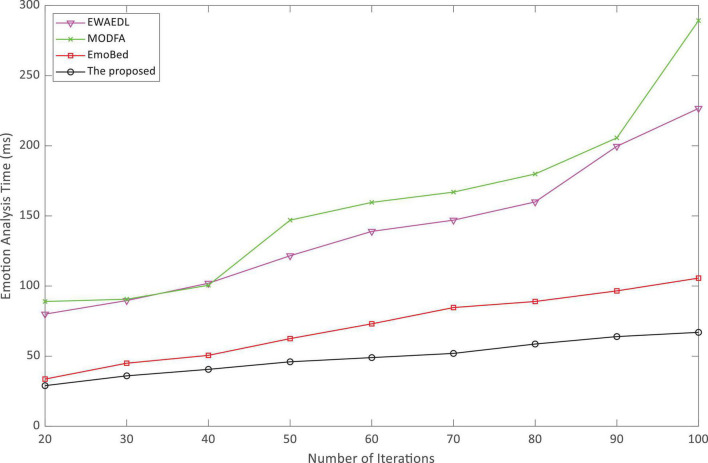
Emotion analysis time of the experiential English teaching.

As indicated in Figure 3, the accuracy of emotion analysis of the algorithm proposed in this paper was always higher than the other three baselines as the increasing number of iterations and basically reached a stable state after the 40th iteration. Throughout the three baselines, the accuracy increased with the increasing of iterations, but it was always below 80%. High accuracy is important for experiential English teaching. Figure 4 shows that the analysis time of the algorithm proposed in this paper is very short, and even at the 100th iteration, it was still less than 100 ms. Surprisingly, EmoBed also had a short analysis time, whereas MODFA and EWAEDL had exponentially increased analysis times. In experiential English teaching, the shorter analysis time provides favorable support for the system, which allows teachers to make English-teaching plans based on the results of EEG analysis.

## Conclusion

Based on the metaverse, this paper designed three periods of student training activities to verify the students’ immersive learning experience. EEG data were captured and analyzed during the students’ experience. Aiming at the difficulty of feature extraction in traditional machine learning and the loss of time-domain information in the CNN emotion recognition process, an EEG analysis based on the CNN–RNN fusion network was proposed. This network combined the advantages of CNN and GRU in extracting frequency-domain and spatial-domain information and achieved high accuracy and short analysis time on the SEED-IV dataset. The final results of the data analysis show that experiential English teaching can improve students’ learning effectiveness, and students’ sense of interaction, immersion, and cognition can be improved.

Professional medical equipment failed to support students to perform free exploration of the educational metaverse, and this technical difficulty is worthy of further research. There are still many deficiencies in this study. At first, due to the limitations of technology, the metaverse-powered experiential situational English teaching in this study still has shortcomings in terms of immersion and needs to be further optimized. In addition, due to the complexity of the operation, the number of students in the experiment is small, and the EEG data and analysis can only reflect the effect of the metaverse-powered experiential situational English teaching to a certain extent. The following research will expand the research scale. Finally, considering the convenience of multi-device collaboration, the EEG monitoring device used in the experiment only had single lead data, and the EEG signals reflected were not comprehensive enough. However, professional medical equipment failed to support students to perform free exploration of the educational metaverse, and this technical difficulty is worthy of further research.

## Data Availability Statement

The raw data supporting the conclusions of this article will be made available by the authors, without undue reservation.

## Ethics Statement

The studies involving human participants were reviewed and approved by Zhejiang Gongshang University. The patients/participants (i.e., undergraduates) provided written informed consent to participate in this study.

## Author Contributions

HG contributed to the methodology, software, and investigation. WG contributed to the conceptualization and writing. Both authors contributed to the article and approved the submitted version.

## Conflict of Interest

The authors declare that the research was conducted in the absence of any commercial or financial relationships that could be construed as a potential conflict of interest.

## Publisher’s Note

All claims expressed in this article are solely those of the authors and do not necessarily represent those of their affiliated organizations, or those of the publisher, the editors and the reviewers. Any product that may be evaluated in this article, or claim that may be made by its manufacturer, is not guaranteed or endorsed by the publisher.
